# Molecular Detection of Bioluminescent Dinoflagellates in Surface Waters of the Patagonian Shelf during Early Austral Summer 2008

**DOI:** 10.1371/journal.pone.0098849

**Published:** 2014-06-11

**Authors:** Martha Valiadi, Stuart C. Painter, John T. Allen, William M. Balch, M. Debora Iglesias-Rodriguez

**Affiliations:** 1 University of Southampton, Ocean and Earth Science, Waterfront Campus, Southampton, United Kingdom; 2 National Oceanography Centre, Ocean Biogeochemistry and Ecosystems, Southampton, United Kingdom; 3 Bigelow Laboratory for Ocean Sciences, East Boothbay, Maine, United States of America; CSIR- National institute of oceanography, India

## Abstract

We investigated the distribution of bioluminescent dinoflagellates in the Patagonian Shelf region using “universal” PCR primers for the dinoflagellate luciferase gene. Luciferase gene sequences and single cell PCR tests, in conjunction with taxonomic identification by microscopy, allowed us to identify and quantify bioluminescent dinoflagellates. We compared these data to coincidental discrete optical measurements of stimulable bioluminescence intensity. Molecular detection of the luciferase gene showed that bioluminescent dinoflagellates were widespread across the majority of the Patagonian Shelf region. Their presence was comparatively underestimated by optical bioluminescence measurements, whose magnitude was affected by interspecific differences in bioluminescence intensity and by the presence of other bioluminescent organisms. Molecular and microscopy data showed that the complex hydrography of the area played an important role in determining the distribution and composition of dinoflagellate populations. Dinoflagellates were absent south of the Falkland Islands where the cold, nutrient-rich, and well-mixed waters of the Falklands Current favoured diatoms instead. Diverse populations of dinoflagellates were present in the warmer, more stratified waters of the Patagonian Shelf and Falklands Current as it warmed northwards. Here, the dinoflagellate population composition could be related to distinct water masses. Our results provide new insight into the prevalence of bioluminescent dinoflagellates in Patagonian Shelf waters and demonstrate that a molecular approach to the detection of bioluminescent dinoflagellates in natural waters is a promising tool for ecological studies of these organisms.

## Introduction

Dinoflagellates are the most ubiquitous protists in the marine environment that produce light [1]–[4], often being responsible for ‘glowing water’ [5] in surface oceanic and coastal waters all over the world [6]–[9]. Light is produced intracellularly in organelles called scintillons [10]. These contain the enzyme luciferase and a luciferin substrate, which in some species is stabilised by a luciferin binding protein [11]–[13]. When cells are mechanically agitated, the luminescent chemistry is activated producing blue light in the form of brief and bright flashes (reviewed by [14]). Bioluminescence in dinoflagellates is thought to have a defensive role against predation, either by using an intense flash of light to startle predators [15] or, according to a more controversial hypothesis [14], [16], by attracting secondary higher level predators which in turn consume the primary predators of bioluminescent dinoflagellates [17], [18]. While bioluminescence may therefore have profound ecological importance across several trophic levels, comparatively little is known about the distribution and ecological characteristics of bioluminescent dinoflagellates.

Ecological studies on bioluminescent dinoflagellates are made difficult by the lack of suitable methods to detect these organisms in mixed planktonic communities. Until now, the only tools to assess the presence and relative abundance of bioluminescent organisms in the water column have been bathyphotometers, instruments that optically measure *in situ* stimulated bioluminescence (henceforth referred to simply as bioluminescence). However, the inlet grid or impeller that stimulates bioluminescence does so indiscriminately in both dinoflagellates and zooplankton, the latter being another important source of bioluminescence. Detailed *in situ* investigations on light producing organisms have shown that both dinoflagellates and zooplankton can both contribute significantly to the stimulated bioluminescence budget depending on the location and season [6], [8], [9], [19]. However, the contribution of each of these groups to a given bioluminescence measurement cannot be readily discerned from bulk bioluminescence measurements.

Dinoflagellate bioluminescence intensity varies both inter- and intraspecifically [20]–[22] and, in some species, it is only detectable at high cell densities [23]. Additionally, bioluminescence only occurs at night, with variable intensity at different times of the diurnal cycle (reviewed by [14]). Environmental and physiological factors can also impact the intensity of bioluminescence produced [24]–[27]. It is therefore unlikely that complex bioluminescent signatures can be used to reveal the distribution of bioluminescent dinoflagellates in diverse oceanic plankton communities. Conversely, gene specific primers designed for the amplification of the luciferase gene (*lcf*) from bacteria [28] and dinoflagellates [23] have detected diverse assemblages of each in natural environments. While the detection of *lcf* only reveals the potential for a cell to produce bioluminescence, we assume that this potential is realized because cells are known to invest considerable resources in bioluminescence, even in long-term culture, and there are no known environmental conditions that suppress the expression of bioluminescence [14], [29]. Therefore, the application of “universal” PCR primers for dinoflagellate *lcf* could be more suitable than existing optical approaches for the study of diverse natural populations of bioluminescent dinoflagellates in ocean waters.

In this study, our aim was to apply “universal” PCR primers for dinoflagellate *lcf* developed using laboratory cultures [29] to detect bioluminescent dinoflagellates in natural populations. To validate our results and place them in an environmental context, we also conducted discrete optical bioluminescence measurements, sequenced *lcf* amplified from environmental samples, characterized the dinoflagellate community by microscopy, and identified bioluminescent species by single cell PCR. We demonstrate that the detection of *lcf* is a promising tool for ecological studies of bioluminescent dinoflagellates.

### Introduction to the Patagonian Shelf

The Patagonian Shelf is located in the southwest Atlantic Ocean along the eastern seaboard of Argentina. It represents one of the most productive regions in the World's oceans and is a globally important CO_2_ sink [30], [31]. The shelf waters are known to harbour diverse assemblages of diatoms and dinoflagellates [32]–[37], including blooms of the bioluminescent species *Alexandrium tamarense* during spring and early summer, particularly along the coast of Argentina [38]–[40].

The hydrography of the Patagonian Shelf and immediate offshore regions (Figure S1) is highly dynamic due to the interaction of subpolar, subtropical and riverine waters derived from the Falklands (Malvinas) and Brazil Currents and the Rio de la Plata outflow, respectively. The Falklands Current is a branch of the Antarctic Circumpolar Current, carrying cold nutrient rich water northwards over the continental shelf slope, until it meets the warm and saline Brazil Current at the Brazil-Falklands Confluence Zone (BFCZ), between 36 and 38°S [41]–[45]. West of the Falklands Current a residual northwards flow parallel to shelf break aligns several water masses in a north-south direction, creating strong zonal gradients in temperature and salinity [46]. Closer to the coast, warmer temperatures and lower salinities signify water inputs from the Pacific through the Magellan Strait [47], [48]. The interaction of shelf waters and the Falklands Current along the steep shelf slope forms the shelf break front. This permanent front is characterised by a pronounced thermal gradient and strong biological productivity [46], [49], [50] supported by upwelling of nutrient rich water [32], [49].

## Methods

### Sample collection

**Figure 1:**
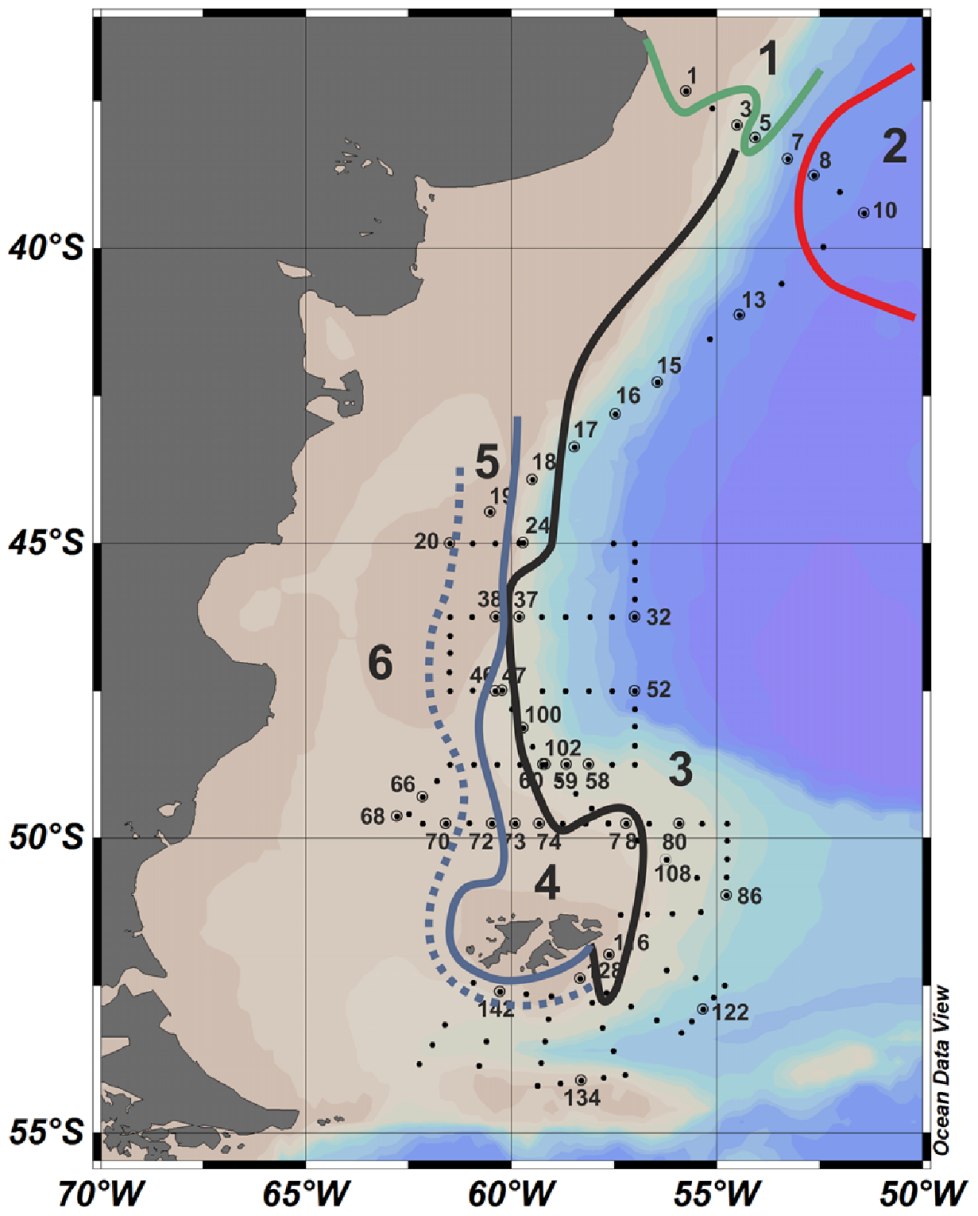
Map of the Patagonian Shelf with sampled stations and distribution of surface water masses. Distinctions between water masses are indicated by coloured lines. These are drawn after Painter et al. [46] but more specifically for the surface depths rather than the whole depth profile. The black line also signifies the position of the shelf break front. The bathymetry is also indicated in the background. 1 =  Rio de la Plata Water; 2 =  Brazil Current Water; 3 =  Falklands Current Water; 4 =  Subantarctic Shelf Water; 5 =  High Salinity Shelf Water; and 6 =  Low Salinity Shelf Water.

Samples were collected during the COPAS '08 (Coccolithophores of the Patagonian Shelf 08) cruise on board the R/V *Roger Revelle*. The cruise took place from the 4^th^ of December 2008 to the 2^nd^ of January 2009 during which several transects were conducted across the Patagonian Shelf break. Forty out of a total of 152 stations were sampled during the cruise (Figure 1), mainly during the night, by a CTD rosette package at surface (1–5 m) and subsurface chlorophyll maxima (SCM) depths. At each sampled depth 20 litres of seawater were gently pre-filtered through 1 mm nylon mesh and collected in darkened carboys to prevent the photoinhibition of bioluminescence. Two litre subsamples were taken for bioluminescence measurements and the rest of the water was filtered onto 12 µm pore size Nuclepore polycarbonate membrane filters (Whatman, UK). For mixed community DNA extractions, filters were immediately frozen at −80°C. For the subsequent isolation of single cells for PCR analysis, cells were rinsed off the filters using 2 mL >99.9% ethanol (molecular biology grade, Sigma, UK) and frozen at −20°C. Subsamples of 100 mL were fixed for microscopy analysis with a Lugols' iodine solution acidified with 10% acetic acid; these samples were initially collected only when a bioluminescence signal was seen but sampling frequency increased after station 16 of the cruise.

### Ethics statement

Permits for all the work described herein were granted from the governments controlling the respective territorial waters (Uruguay, Argentina and Great Britain) to the *R/V Revelle* and communicated through the State Department of the U.S.A. International observers accompanied the cruise and cruise data was given to each of the countries. No protected species were sampled as part this work.

### Bioluminescence measurements

Bioluminescence was measured using a Glowtracka bathyphotometer (Chelsea Technologies, UK) that had been converted for benchtop use and was able to process 2 L discrete samples. The setup consisted of a 2 L reservoir attached to a pipe leading into the measuring chamber. The water was kept in the chamber and reservoir by a closed tap at the outlet of the instrument. Stimulation of bioluminescence was achieved using a 1 mm nylon mesh at the entrance of the detection chamber. This setup was tested in the lab prior to its use at sea using dilute large-volume cultures of *Lingulodinium polyedrum* and *Pyrocystis lunula*, to optimize the mesh size and sample volume. A later improved version of this setup has been described by Marcinko et al. [51].

Bioluminescence measurements commenced after allowing the samples to stand for 15 minutes so that bioluminescence could partially recover and flashing induced by turbulence in the sample could cease. Two litres of seawater were accurately measured using a measuring cylinder, gently poured into the reservoir of the instrument and left to settle following handling for another 5 minutes. To ensure comparability of the samples, these timings were kept precise so that any pre-stimulation of bioluminescence was identical. The tap that held the water in the reservoir was then released allowing the water to flow through the instrument. Bioluminescence was measured every millisecond for approximately 15 seconds in the form of a voltage signal. This was recorded by a data logger controlled by LabView software (National Instruments, UK). Two litres of fresh water were run through the instrument between every sample measurement to clean the instrument of residual cells and to obtain a baseline measurement. A typical signal obtained from the instrument and details of the data processing are shown in Figure S2. The bioluminescence intensity data obtained during this cruise are provided in Table S1.

### DNA extractions

The frozen samples of filtered plankton were processed directly on the filters. Cells were ruptured by immersing the tubes containing the filters in liquid nitrogen and grinding the frozen cells on the filter using a micropestle until they began to thaw; this procedure was repeated three times. Further rupture of cells was achieved by the addition of 300 µL boiling buffer (1.4 M NaCl, 100 mM Tris HCl, 20 mM EDTA) and incubation at 90°C for 10 minutes. This step was important for disrupting ‘tough’ organisms such as *Ceratium* spp. and was found to increase the DNA yield up to 5-fold. Cells were lysed by incubation at 65°C for 1 hour in pre-warmed cetyl- trimethylammoniumbromide (CTAB) buffer (2% CTAB, 2% polyvinylpyrrolidone (PVP), 0.5% 2-mercaptoethanol, 1.4 M NaCl, 20 mM EDTA and 100 mM Tris HCl) achieved by adding an equal volume (300 µl) of this lysis buffer with double concentrations of CTAB, PVP and 2-mercaptoethanol to the boiling buffer containing the ruptured cells. The rest of the extraction followed the protocols described in Valiadi et al. [29]. The DNA was dissolved in 30 µL of TE buffer (10 mM Tris and 1 mM EDTA), its purity and quantity were measured using a Nanodrop ND-3000 spectrophotometer (Nanodrop, U.S.A.) and its PCR quality was assessed by amplification of eukaryotic small subunit ribosomal DNA using primers Euk1A/Euk516r-GC [52]–[54].

### Preparation of cells of single cell PCR

At least 3 cells of each *Ceratium* species present in our sample set were subjected to PCR. This analysis focused on this genus because several of its species were abundant in the region and there is great uncertainty over which of these are bioluminescent [29]. Cell suspensions stored in ethanol were concentrated by gentle centrifugation at 4000 g, the supernatant was discarded and replaced with TE buffer. This was repeated three times to thoroughly remove the ethanol. Individual cells identified under an inverted microscope were isolated in 1 µL TE buffer using a micropipette and then transferred to a PCR tube. Cells that were not used immediately were stored at −80°C.

### Detection, cloning and sequencing of the luciferase gene

Dinoflagellate *lcf* was amplified using primers DinoLCF_F4 and DinoLCF_R2 and the protocol described by Valiadi et al. [29]. The templates for the PCR were either 1 µL of extracted DNA (maximum 1 µg), or a single cell in 1 µL TE buffer that had been disrupted immediately prior to the addition of the PCR components by boiling at 90°C for 10 minutes followed by rapid cooling on ice. To confirm that the correct gene was amplified the PCR bands from 10 of the mixed community DNA samples were purified from agarose gels and cloned into the pCR 4 vector in the TOPO TA cloning kit (Invitrogen, USA). These samples represented stations with four observational scenarios: high bioluminescence and high bioluminescent dinoflagellate concentration (stations 1 and 5 SCM and stations 46 and 47 surface samples), high bioluminescence and low bioluminescent dinoflagellate concentration (stations 68 and 134 surface samples), low bioluminescence and low/no bioluminescent dinoflagellate concentration (station 17 SCM, 24 and 74 surface samples) and low bioluminescence and high bioluminescent dinoflagellate concentration (station 80 surface sample). Four clones were sequenced from each of these samples using the M13 forward primer by Source Bioscience (UK). All sequences have been submitted to GenBank (accession numbers KF735135-77).

### Sequence analyses

The sequences obtained from the clones of the *lcf* PCR products were trimmed of vector and primers and their identity was confirmed using the BLASTn tool of the NCBI database (http://blast.ncbi.nlm.nih.gov/Blast.cgi). All further sequence analyses were conducted in the MEGA v. 5 software [55]. New sequences were aligned to those of cultured representatives obtained from GenBank that had been split into the three repeated domains that comprise the *lcf* in photosynthetic species. Alignments were carried out using ClustalW [56] and improved manually. A genetic distance (p-distance) matrix was generated to compare the similarities of environmental *lcf* sequences to those of cultured isolates. Sequences of different lengths were compared by discarding gaps only in pairwise comparisons. The distance matrix was visualized as a dendrogram constructed using the Unweighted Pair Group Method with Arithmetic Mean (UPGMA) algorithm and statistically assessed with 1000 bootstrap replications.

### Microscopy

Samples fixed in Lugol's iodine containing 10% glacial acetic acid were examined under an inverted light microscope (X200; Brunel microscopes, U.K.) using the Utermöhl method [57]. A 50 mL aliquot was left to settle overnight and cells were enumerated at 100× or 200× magnification depending on their size and density in the sample. Cells larger than approximately 8 µm were identified according to Hasle and Syvertsen [58] and Steidinger and Tangen [59].

### Nutrient and chlorophyll measurements

The macronutrients nitrate, phosphate, silicic acid and ammonium were measured using an autoanalyser and standard protocols [60]; data were kindly provided by Dan Schuller (Ship Operations & Marine Technical Support Services, Scripps Institution of Oceanography, California, USA). For chlorophyll analyses, 200 mL subsamples were filtered onto GF/F filters (Whatman) and extracted in 10 mL 90% acetone at −20° for 12 h [61]. Fluorescence was measured using a Turner Designs AU-10 fluorometer calibrated against a chlorophyll *a* standard (Sigma Aldrich). Cruise data have been deposited at the Biological & Chemical Oceanography Data Management Office (BCO-DMO) and can be accessed at the following link: http://www.bco-dmo.org/project/2074.

## Results

### Environmental setting

The study area consisted of a number of hydrographic provinces, which have been described in detail for this cruise by Painter et al. [46]. The cruise track annotated with sampled station numbers and the position of six key water masses relevant to this study and presented previously by Painter et al. [46] are shown in Figure 1; the characteristics of each water mass is summarised in Table 1. At the northern end of the study region, north 40°S, surface waters were dominated by Rio de la Plata Water (stations 1 and 5, west of 54.07°W) and southward flowing Brazil Current Water (stations 8–11, east of 52.63°W). The remainder of the cruise repeatedly crossed the northward flowing Falklands Current Waters and the Shelf Waters to the west. The transition between Shelf Waters and Falklands Current Waters signified the position of the shelf break front. Shelf Waters were further subdivided from east to west into Subantarctic Shelf Water, High Salinity Shelf Water and Low Salinity Shelf Water, which all extended parallel to one-another in a north-south direction [46].

**Table 1:** Characteristics of surface water masses.

Water mass	Salinity	Temperature
Rio de la Plata outflow	<33	17–18
Brazil Current Waters	>34	16–19
Falklands (Malvinas) Current Water	>33.9	-
Shelf Water: Subantarctic	33.78–33.9	-
Shelf water: High salinity	33.58–33.78	-
Shelf water: Low salinity	<33.58	-

The temperature and salinity characteristics of water masses are based on Painter et al. [46] but where necessary modified to apply to the surface depths that relate to our study.

**Figure 2:**
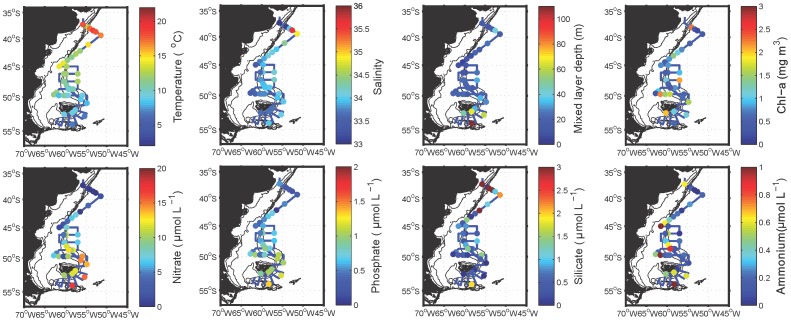
Surface distribution of physical and chemical variables. Maps of the Patagonian Shelf showing the large scale surface distributions of physical and chemical variables relevant to this study. Data shown are from the whole cruise dataset.

The surface distributions of some key physical and chemical variables as well as the mixed layer depth are shown in Figure 2. These data, except for discrete chlorophyll *a* measurements, were previously presented in Painter et al. [46] and for consistency with that study, the mixed layer depth was defined as the depth where temperature decreased by 0.5°C relative to surface values [62]. The area south of the Falkland Islands (i.e. south of approximately 52°S) was characterised by colder waters (<10°C) and a deeper mixed layer (>50 m). As these waters flowed northwards, surface temperature increased to approximately 13°C complemented by a shoaling of the mixed layer depth to 20–30 m. East-west gradients in temperature were not pronounced even though the salinity data clearly showed the distinction between the Shelf (S<33.9) and Falkland Current Waters (S>33.9).

Nitrate and phosphate concentrations followed a similar latitudinal gradient to temperature with high surface nitrate (>10 µM) and phosphate concentrations (>0.8 µM) south of the Falkland Islands, which declined rapidly northwards in both the Shelf and Falkland Current Waters. Despite the latitudinal decline, concentrations generally remained high (approximately 2 µM nitrate and 0.2 µM phosphate) across most of the study area until low nutrient waters of the Brazil Current were encountered north of 40°S. In the northern area of the study region, waters from the Rio de la Plata and from the Brazil Current were both poor in surface macronutrients (<0.01 µM nitrate and <0.3 µM phosphate) but had a high silicate load (3–14 µM), particularly near the Rio de la Plata outflow. Otherwise, silicate concentrations were generally low (<2 µM) in the rest of the study region. Similarly, ammonium concentrations, although patchy, decreased northwards. East-west gradients were evident in most macronutrients, particularly nitrate and phosphate, with lower nutrient concentrations to the west of the shelf break front within Shelf Waters, particularly in the Low Salinity Shelf Water where nitrate was depleted. Additionally, nitrate, phosphate and ammonium were higher near the shelf break front between the latitudes 47–50°S, compared to adjacent areas. Chlorophyll *a* concentrations generally ranged from 0.1–3.9 µg L^−1^ (Figure 2 and Table S1) and showed a patchy distribution not corresponding to trends in nutrient concentrations. Some of the highest chlorophyll *a* concentrations were observed at stations near the shelf break front (e.g. stations 5, 18, 46, 60 and 74).

### Luciferase gene detection in mixed communities

The amplification of *lcf* gene from natural samples was highly specific producing well-defined PCR bands without non-specific background amplification (Figure S3). Of the 72 samples analysed (after three being omitted due to poor purity and PCR failure with general primers for eukaryotes), 47 produced a PCR product corresponding to the expected 270 bp band for *lcf*. The specificity of the primers to the correct gene was confirmed by sequencing 40 clones from 10 samples, representing approximately 20% of the samples that produced PCR product.

### Distribution of luciferase and bioluminescence

**Figure 3:**
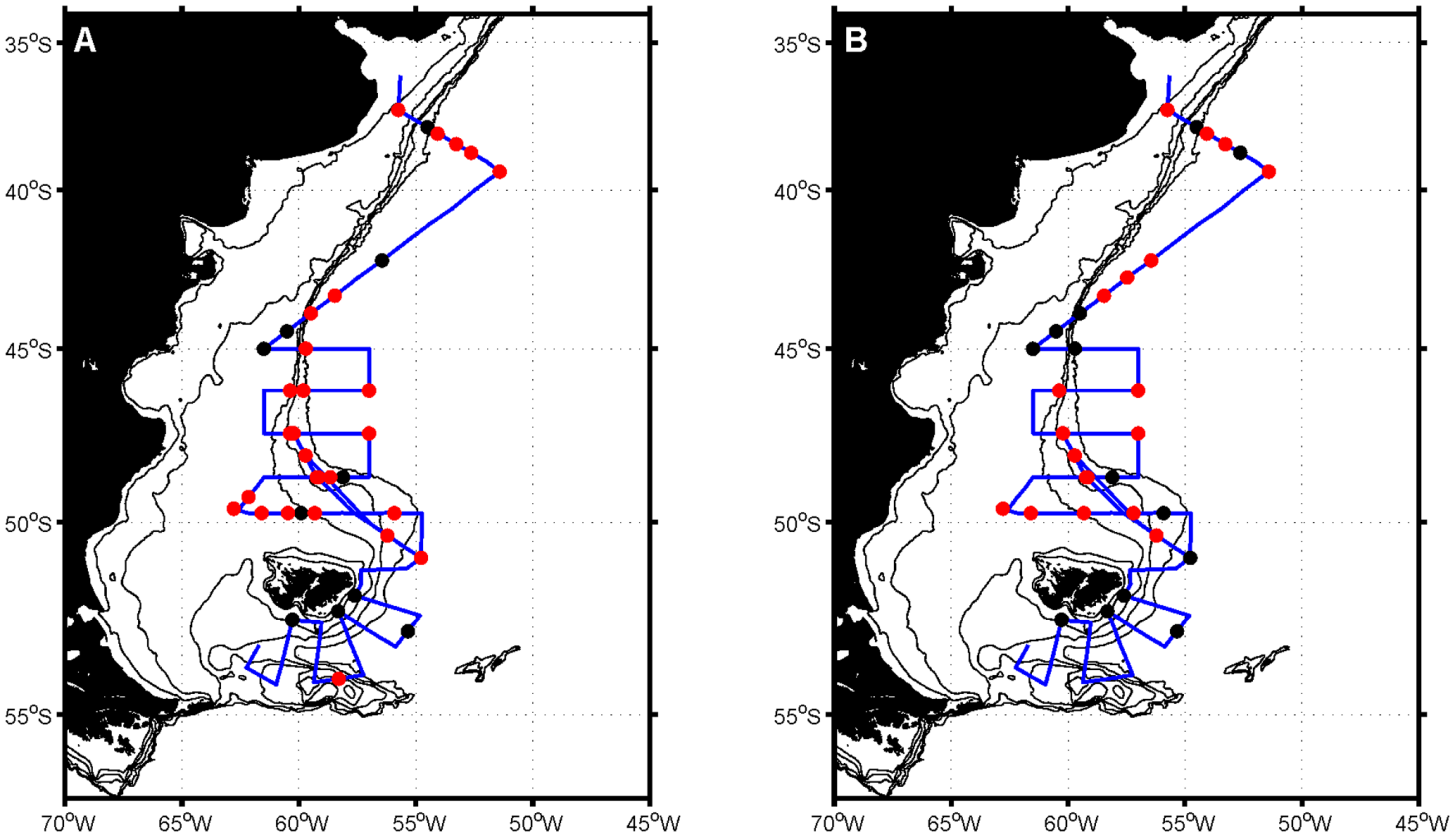
Distribution of the luciferase gene. Maps of the Patagonian Shelf showing the distribution of the luciferase gene at surface (A) and chlorophyll maximum depths (B). Red circles indicate that luciferase was detected while black dots indicate that luciferase was not detected. Note that results from some closely spaced stations overlap.

The presence of *lcf* (Figure 3) was widespread in most of the study region with 65% (47 of the 72) of the samples analysed containing this gene. A marked absence of *lcf* was evident south of the Falkland Islands with only one surface sample at station 134 being positive for this gene. In addition, *lcf* was more frequent in surface waters than at the subsurface chlorophyll maximum with 73% (27 of 37) and 54% (18 of 33) of samples containing *lcf* in the former and the latter, respectively.

**Figure 4:**
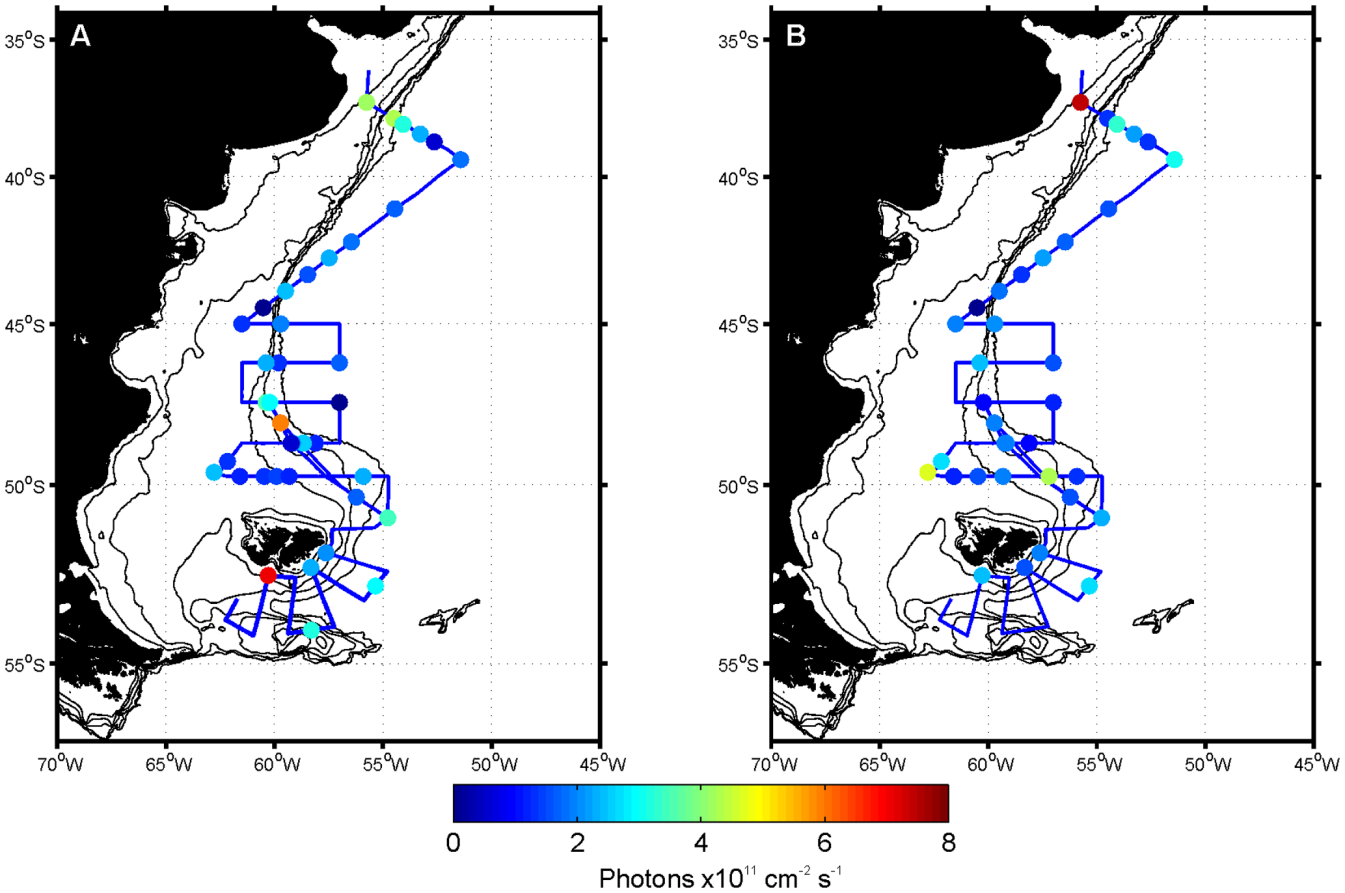
Distribution of bioluminescence. Maps of the Patagonian Shelf showing the distribution of bioluminescence at A) surface and B) chlorophyll maximum depths. Note that values below 2.5×10^11^ photons cm^−2^ s^−1^ (i.e. blue shades) are below the detection limit and that some stations overlap.

Bioluminescence intensity above the detection limit was only measured in 13 samples (Figure 4). Detectable bioluminescence was found in the northernmost stations, some stations near the shelf break front and in surface waters of the southernmost stations. The highest bioluminescence values, above 5×10^11^ photons cm^−2^ s^−1^, were recorded in the SCM sample of station 1 and the surface samples of stations 46, 47 and 142. Both the horizontal and vertical distributions of bioluminescence were patchy and bioluminescence could be absent at the surface but present in the deep sample, or vice versa, depending on location.

### Characterization of bioluminescent dinoflagellate populations

**Figure 5:**
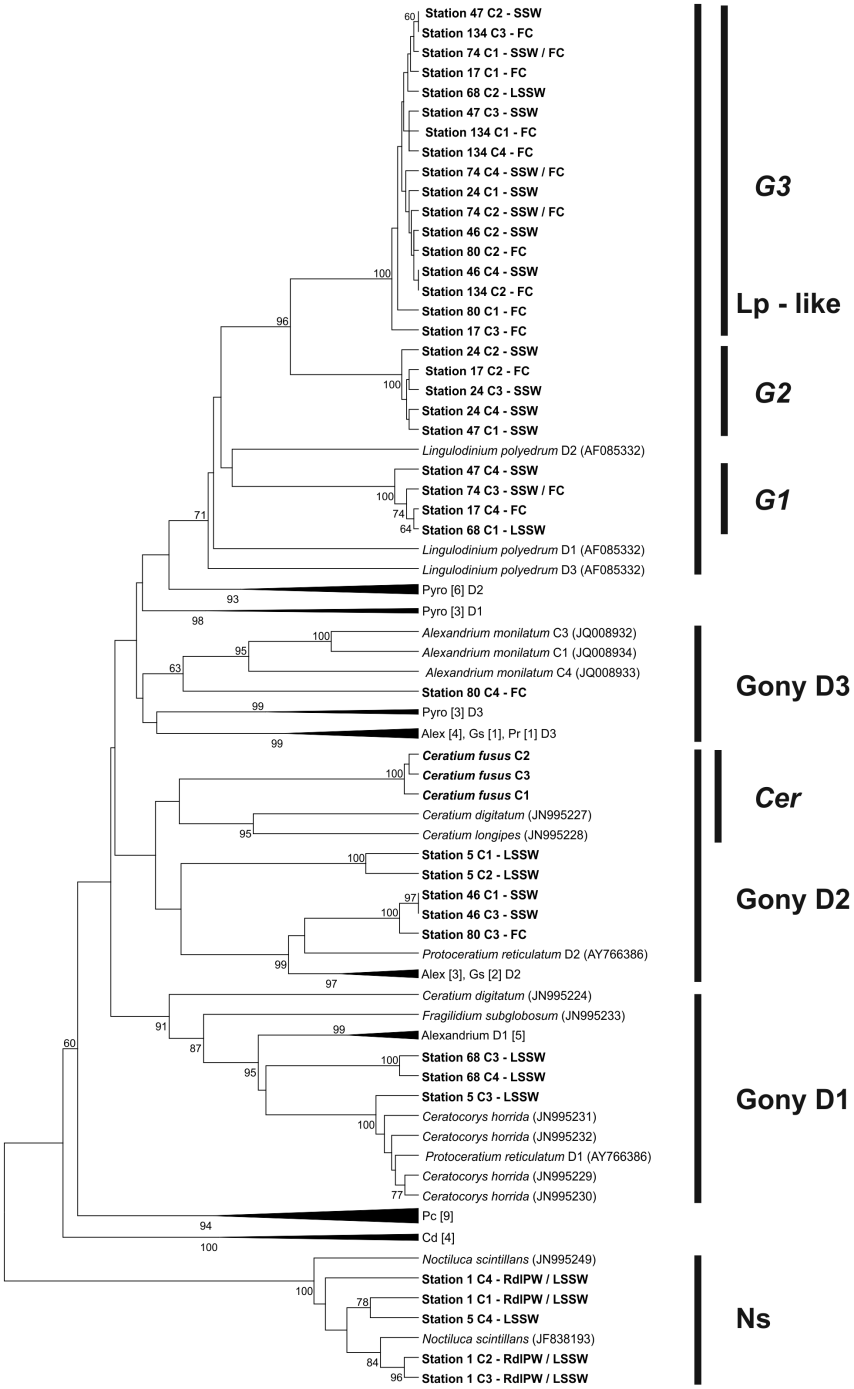
Dendrogram of *lcf* sequences. Sequences amplified from selected stations and those from GenBank were analysed using the Unweighted Pair Group Method with Arithmetic Mean (UPGMA) based on genetic distance (p-distance). Important groups are labelled with vertical bars. Major groups are in bold font and subgroups in bold italics. Bootstrap values (>60%) are shown at the nodes. Taxon abbreviations: Alex  =  *Alexandrium*, C =  Clone, Cer  =  *Ceratium*, Cd  =  *Ceratium digitatum*, D =  Domain, Gony  =  *Gonyaulacales*, Gs  =  *Gonyaulax spinifera*, Lp  =  *Lingulodinium polyedrum*, Pc  =  *Protoperidinium crassipes*, Pr  =  *Protoceratium reticulatum*, Pyro  =  *Pyrocystis*. Water mass abbreviations: FC  =  Falklands Current Water, HSSW  =  High Salinity Shelf Water, LSSW  =  Low Salinity Shelf Water, RdlPW  =  Rio de la Plata Water, SSW  =  Subantarctic Shelf Water. When a branch is collapsed the number of sequences from each group within it is indicated in square brackets.

Four *lcf* sequences were obtained from each of the 10 samples selected for sequencing, resulting in a total of 40 sequences. Hierarchical clustering was used to match sequences to their most similar cultured representatives (Figure 5). Sequences with 92–97% identity to *Noctiluca scintillans* were only detected at stations 1 and 5 in waters influenced by the Rio de la Plata, making up all the sequences obtained from station 1. The rest of the samples from the Shelf Waters were dominated by sequences from an organism that was most similar to *Lingulodinium polyedrum* (Lp-like group, 83–85% identity), accounting for 26 out of the 35 remaining sequences. These sequences formed 3 distinct subgroups (G1–G3) with differing levels of similarity to *L. polyedrum* but with unassigned genus. A few sequences from stations 5, 37 and 60 also showed similarities to *Alexandrium tamarense*, *Ceratocorys horrida*, *Gonyaulax spinifera* and *Protoceratium reticulatum*.

**Figure 6:**
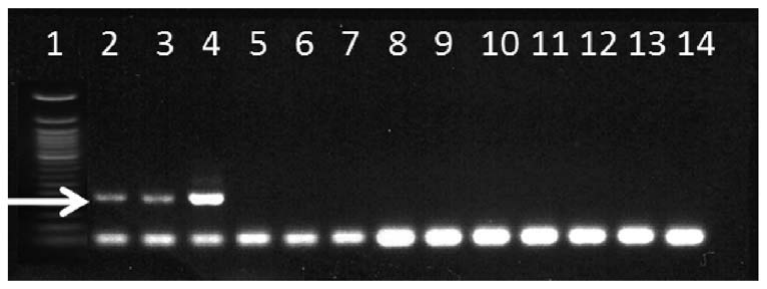
Single cell PCR tests. Gel photograph of representative PCR tests for the detection of the luciferase gene in single cells of various *Ceratium* species. Lane contents: 1) 50 bp DNA marker; 2–4) *Ceratium fusus*; 5–7) *Ceratium furca*; 8–10) *Ceratium tripos*; 11–12) *Ceratium lineatum*; 13–14) *Ceratium* cf. *teres*. The arrow indicates the amplified 270 bp fragment.

The PCR assay was also applied to single cells in order to distinguish bioluminescent from non- bioluminescent species in the genus *Ceratium* (Figure 6). Three cells of *Ceratium fusus* produced a PCR band, being the only ones containing *lcf*. In contrast, *lcf* was not found in *C. furca*, *C. lineatum*-like, *C. tripos* and a *C. teres*-like species. Sequences of *C. fusus lcf* were most similar to other species from the genus.

Based on these analyses and literature reports [29], [63] we separated dinoflagellates into bioluminescent and non-bioluminescent groups. The bioluminescent group consisted of *Gonyaulax*-like dinoflagellates (including *Alexandrium*, *Lingulodinium* and *Protoceratium* species), *C. fusus*, *Noctiluca scintillans*, *Protoperidinium* spp., *Pyrocystis* spp. and *Pyrophacus* sp. Other dinoflagellates that were present were mainly of the genus *Prorocentrum* and the order Gymnodiniales along with some *Dinophysis* spp.; all these dinoflagellates were classified as non-bioluminescent. Using these data we also estimated the sensitivity of the *lcf* PCR assay. Detection of *lcf* was sometimes more sensitive than microscopy (e.g. station 5 SCM and 24 surface), detecting *lcf* where no bioluminescent dinoflagellates were counted. When more than 900 bioluminescent cells (based on microscopy) were present in the sample, *lcf* detection and microscopy observations agreed.

### Comparison of bioluminescence measurements to luciferase gene detection and bioluminescent dinoflagellate cell counts

**Figure 7:**
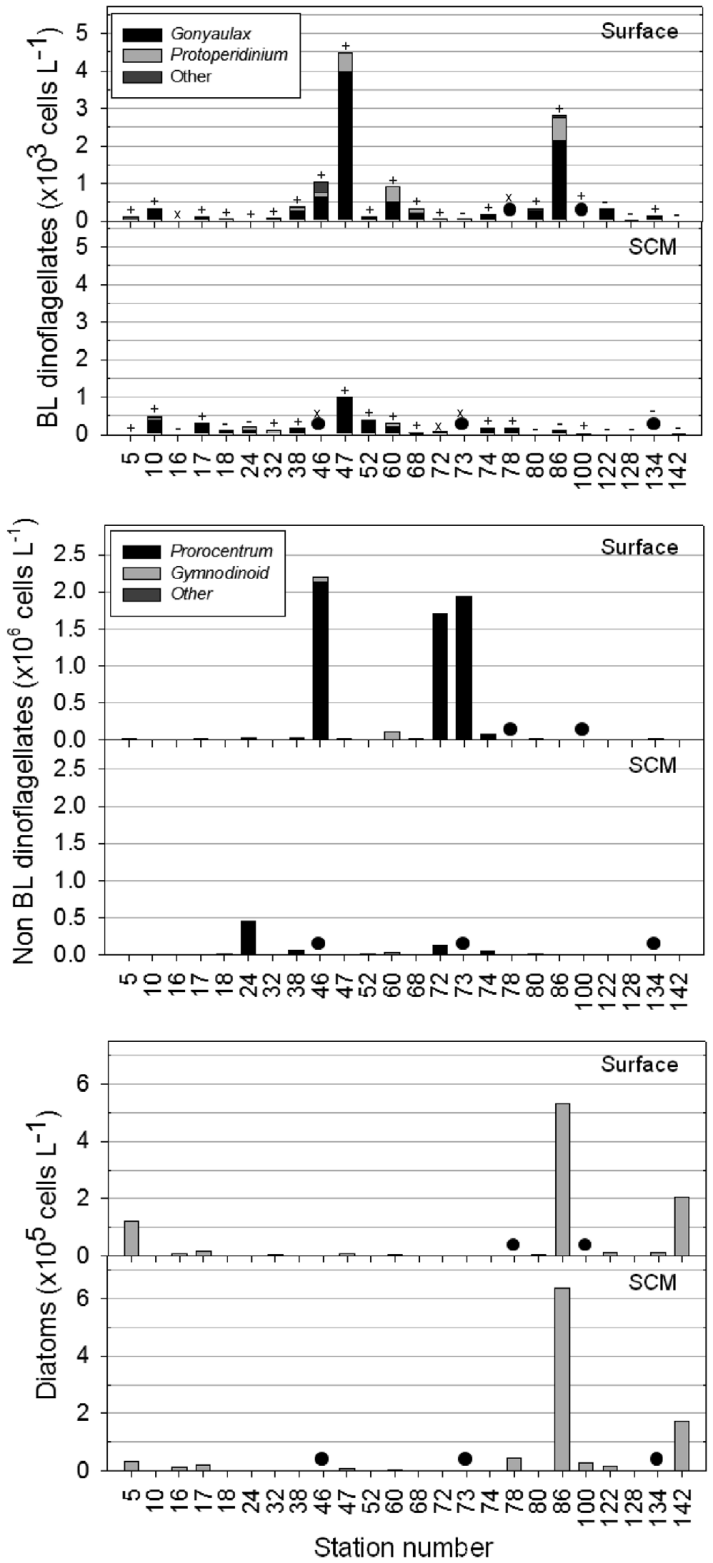
Abundances of key protist groups. Data are from surface depths (upper panel) and at the depth of the subsurface chlorophyll maximum (SCM; lower panel), at the stations sampled in this study. A black circle indicates no data at that point rather than a zero value. *Lcf* detection results are superimposed on the plot of bioluminescent dinoflagellates showing positive (+), negative (−), or missing data (x) for each sample.

Relative to the detection of *lcf* (Figure 3), the number of samples containing bioluminescent dinoflagellates was comparatively underestimated at least 3-fold by bioluminescence measurements (Figure 4). Additionally, two samples where bioluminescence was detected, did not correspond to a positive detection of *lcf* (stations 3 and 142 surface) and another four samples were associated with very low concentrations (<0.2×10^3^ cells L^−1^) of bioluminescent dinoflagellates (Figure 7; stations 134 surface and 5, 60 and 78 subsurface chlorophyll maximum).

Bioluminescence intensity did not correlate with the number of bioluminescent dinoflagellate cells present (Spearman's ρ = −0.607, p>0.1, n = 7). This was despite the exclusion of stations where bioluminescence was detected but *lcf* was not and stations where bioluminescence was below the limit of detection, which did however result in a limited dataset. Nevertheless, underlying spatial differences in community composition may provide an explanation for this. For example, although a similar amount of light (3–3.5×10^11^ photons cm^−2^ s^−1^) was measured in the surface waters of stations 5 and 46 bioluminescent cell densities differed by an order of magnitude (120 cells L^−1^ and ∼1000 cells L^−1^, respectively; Figure 7). At these stations the bioluminescent dinoflagellate population was dominated by different organisms, with *N. scintillans* dominant at station 5 and *Gonyaulax* spp. at station 46. In another example, surface bioluminescence was less intense at station 47 than station 46, even though the former contained a 4-fold higher number of bioluminescent *Gonyaulax*-like dinoflagellates. Critically in this example, different sampling times during the diel cycle may explain the difference with station 46 measured at 22.30 and station 47 at 03.00.

### Distribution and composition of dinoflagellate populations identified by microscopy

Microscopy analyses (Figure 7) revealed that dinoflagellates were generally absent in waters south of the Falkland Islands, which instead contained diatom populations. Diverse populations of dinoflagellates were present to the north of the Falkland Islands and were generally more abundant at the surface than at the subsurface chlorophyll maximum. The highest concentration of dinoflagellates (>2 million cells L^−1^) was found at station 46 near the shelf break front coincident with the highest chlorophyll *a* concentration of nearly 4 µg L^−1^ (Figure 2). Bioluminescent cell abundances, which were well below bloom densities (<100,000 cells L^−1^) throughout the study region, were dominated by gonyaulacoid dinoflagellates. Most of these cells belonged to the genus *Gonyaulax* and less frequently to other genetically similar genera (e.g. *Alexandrium* and *Protoceratium*, Figure 5). These species together composed the dominant *Gonyaulax*-like group, which was responsible for the highest abundances of bioluminescent cells, including a maximum of ∼4000 bioluminescent cells L^−1^ at station 47.

Non-bioluminescent dinoflagellates were generally present at much higher abundances across the Patagonian Shelf than bioluminescent dinoflagellates (Figure 7). The numerically most abundant species was *Prorocentrum* sp., which formed a pronounced bloom along the shelf break front in surface waters at stations 46, 72 and 73. This species was present at concentrations of more than 1.5 million cells L^−1^ at these stations, which resulted in visible discoloration of the water. High abundances of *Prorocentrum* sp. were spatially distinct from stations where the maximum abundance of *Gonyaulax* spp. were found (station 46 versus 47; Figure 7). Nevertheless, both species were generally found in Subantarctic Shelf Waters (Figure S1), but neither displayed a clear relationship to nutrient concentrations (Figure 2).

Warmer waters north of 40°S (Figure 2) supported distinct dinoflagellate assemblages. Both microscopy data and *lcf* sequences (Figure 5) indicated that low nutrient and salinity waters influenced by the Rio de la Plata outflow (Figure 1) contained *N. scintillans* as the main bioluminescent dinoflagellate; stations that also had high bioluminescent intensities (Figure 4). Non-bioluminescent *Ceratium* also reached maximum abundance in this region (∼12000 cells L^−1^), mainly due to *C. tripos*. Waters associated with the oligotrophic and saline Brazil Current sampled at station 10 (Figure 1) were dominated by *Alexandrium* spp., representing the only station where this genus was a significant component of the protist community.

## Discussion

### Detection of the luciferase gene in natural waters

The application of primers specific and “universal” to dinoflagellate *lcf* to natural water samples from the Patagonian Shelf region has provided a unique and novel view of the distribution of bioluminescent dinoflagellates in this region. The discovery that bioluminescent dinoflagellates are widespread, although not highly abundant in the area was made possible due to the high sensitivity of the *lcf* detection technique. The variation in the estimated sensitivity of the PCR protocol (0–900 cells) is most likely due to a varying number of domains contained (or amplified) in the *lcf* of different organisms [13], [29]. Nevertheless, even the largest number of cells (900) corresponds to a cell density of 0.3 cells mL^-1^ which is comparable to the detection limits of other dinoflagellate targeted PCR protocols [64]–[66].

Identifying the dinoflagellate species that produce bioluminescence in the water column by microscopy alone is not straightforward as there are several species whose bioluminescence potential is ambiguous [29]. In this study robust classification of the bioluminescent and non-bioluminescent dinoflagellate groups was enabled by the identities of *lcf* sequences obtained from mixed community samples and by the identification of *lcf* in single cells of *Ceratium* spp. The genus *Ceratium* was common in the study area and is known to contain a few bioluminescent species but many are largely un- or mischaracterised [29]. For example, *C. furca* has been reported as bioluminescent [63] but was found here not to contain *lcf*, confirming earlier observations of this species as being non-bioluminescent [67]. *Ceratium fusus* however, was found to contain *lcf*, in agreement with previous studies from the North Atlantic [8] and California [67]. The genus *Protoperidinium*, which was included in the bioluminescent group, is also known to contain bioluminescent and non-bioluminescent species, but the group was not widely distributed in the area and its contribution to the bioluminescent field is considered minimal. In fact, the only taxon that was abundant and could easily be assigned to the bioluminescent group was *Gonyaulax* spp. and morphologically similar species which are mostly bioluminescent [29], [63]. This taxon also dominated the *lcf* sequences retrieved from mixed community samples. Non-bioluminescent dinoflagellates in the dominant genus *Prorocentrum* as well as members of the Gymnodiniales were easier to categorise due to strong evidence for the lack of bioluminescence and *lcf* in these species [29]. In summary, the molecular detection of *lcf* in mixed dinoflagellate communities and in single cells enabled accurate identification of bioluminescent and non-bioluminescent dinoflagellates providing considerably more insight into the ecology of these groups compared to bioluminescence intensity measurements alone.

### Drawbacks of using optical bioluminescence measurements in ecological studies of bioluminescent dinoflagellates

The data produced in this study provide the first opportunity to compare optical bioluminescence measurements with corresponding molecular (bioluminescence capability) and microscopic data (species information) in order to assess the usefulness of optical bioluminescence measurements alone in ecological studies of bioluminescent dinoflagellates. We found that bioluminescence measurements underestimated the presence of bioluminescent dinoflagellates more than 3-fold relative to the detection of *lcf*. The optical detection of bioluminescence therefore limits our ability to map the distribution of bioluminescent dinoflagellates. Furthermore, even when bioluminescence intensity was high the observed magnitude was likely affected by other organisms (e.g. zooplankton) and by intraspecific variability in the bioluminescence properties of dinoflagellates, such that simple correlations between bioluminescence intensity and dinoflagellate abundance cannot be substantiated. In samples where bioluminescent dinoflagellates were completely or nearly undetectable by PCR or microscopy techniques, we assume that any observed bioluminescence was attributable to zooplankton. It is though, not possible to deconstruct a bioluminescence measurement into the constituent zooplankton and dinoflagellate parts. Moline et al. [68] used a rough correlation between size and flash intensity of various bioluminescent organisms to distinguish the flashes of dinoflagellates from those of larger zooplankton. However, their data showed a considerable overlap in flash intensity between dinoflagellates and zooplankton groups in the small (<1 mm) size range that was targeted in the present study making this approach inapplicable here.

In samples where bioluminescence likely originated only from dinoflagellates, the measured intensity was very likely to be affected by both interspecific differences in flash intensity and by cellular diel rhythms. For example, *N. scintillans* which is known to produce a high intensity flash [69] was detectable at a concentration of 120 cells L^−1^ at station 5, while the detection of the dimmer *Gonyaulax* spp. [8], [23] required 10× greater cell abundance (∼1000 cell L^−1^). Thus, to a large extent the spatial variability in bioluminescence intensity is directly related to dinoflagellate population composition.

In addition to population composition, the presence of a diel rhythm in dinoflagellates is increasingly recognised as an important variable in bioluminescence field studies. For example, observations of a diel rhythm of bioluminescence within a mixed dinoflagellate community from the North Atlantic [51] suggest that only bioluminescence measured at the same time of night can be used to accurately monitor changes in bioluminescent intensity related to the environment. The results of the present study additionally show that interspecific differences in the magnitude of bioluminescence mean that bioluminescence measurements are only comparable when the species composition is constant, a situation that is likely to be encountered only within monospecific dinoflagellate blooms. Therefore, in order to use optical bioluminescence measurements as a tool to monitor bioluminescent dinoflagellate populations, or to ensure that bioluminescence datasets are comparable, three conditions must be met: 1) no zooplankton must be present, 2) only measurements collected at the same time of night can be compared, 3) the composition of the population must be constant. Such conditions are highly prescriptive and unlikely to be achievable in the field, making the alternative use of sensitive molecular techniques to describe natural mixed populations highly desirable.

### Environmental structuring of bioluminescent dinoflagellate populations

Molecular and microscopic analyses confirmed that environmental conditions were important in driving the distribution and composition of both bioluminescent and non-bioluminescent dinoflagellate populations. Large-scale features such as the absence of dinoflagellates from the cold well-mixed waters in the south of the study area were readily apparent from both the PCR of *lcf* and from cell counts. To the north of the Falkland Islands conditions were more favourable for dinoflagellates and included higher temperatures typical of temperate latitudes (13–22°C), a relatively shallow mixed layer depth (<30 m) and generally low but adequate macronutrient concentrations (approximately 2 µM nitrate and 0.2 µM phosphate). Silicate concentrations were potentially limiting for large diatoms [70], [71], which may have provided the environmental niche needed for dinoflagellates to successfully compete in these waters. However, a bloom composed of coccolithophores and small diatoms (<10 µm) was present in the Falklands Current [72], although it did not overlap with stations of high dinoflagellate abundance.

The physical and chemical properties of the region appear to be critical in determining the location, composition and abundance of dinoflagellate populations. Waters north of 40°S that were composed of waters from the Rio de la Plata outflow and subtropically derived Brazil Current Waters were physically and chemically distinct to the rest of the study area. In the Rio de la Plata outflow waters, both cell counts and *lcf* sequences revealed that *N. scintillans* was the main dinoflagellate responsible for bioluminescence, followed by *C. fusus*. Both species together with the non-bioluminescent *Ceratium* spp. represent a typical seasonal community in these waters [35], [36]. Stations located within the influence of the Brazil Current supported a different dinoflagellate population dominated by *A. tamarense,* although the oligotrophic conditions of these waters [46], [73], [74] only allowed for a low abundance of these cells.

The dinoflagellate populations in the area between the Falkland Islands and 40°S were composed of genetically closely related gonyaulacoid dinoflagellates that were responsible for the highest abundances of bioluminescent dinoflagellates at the shelf break front. The species present were mainly of the genus *Gonyaulax* according to microscopy, and *Lingulodinium polyedrum*-like, according to *lcf* sequences. Within these populations there was no specific pattern of association of certain genotypes or morphotypes to specific water masses. Therefore, the four water masses that can be distinguished by subtle changes in salinity in this area (Falkland Current Waters and three types of Shelf Waters, Table 1) were not dissimilar enough to cause any significant shifts in the dinoflagellate population composition or distribution. This suggests that the waters of the central shelf may be conducive to dinoflagellate dominance. Contrary to previous reports however, we found that the abundance of *A. tamarense* was low even at the most inshore stations suggesting that its dominance may be restricted to near-coastal areas [38]–[40] either by the residual northward advective flow of the shelf region or by the presence of several hydrographic fronts that are present across the shelf [46].

Upwelling along the shelf break front is highly likely to supply essential macronutrients and shelf derived iron to surface waters that are essential for the maintenance of the persistent phytoplankton bloom that forms along this front throughout the spring and summer [32], [49]. We found that the shelf break front supported both bioluminescent and non-bioluminescent dinoflagellates species, and maximum abundances of both *Gonyaulax* spp. and *Prorocentrum* sp. were found here although at slightly differing locations (e.g. station 46 versus 47). Our observations of elevated macronutrient and chlorophyll *a* concentrations at stations situated in the shelf break front are coincident with an exceptionally intense *Prorocentrum* sp. bloom at station 46. However, at several other stations near the shelf break front (e.g. stations 60 and 74) high chlorophyll *a* was not associated with dinoflagellates or diatoms but most likely with a declining coccolithophore bloom [46]. This suggests that the shelf break front represents a unique environment that is important for several phytoplankton functional groups at different stages of the population succession.

## Conclusions

This study describes the first application of a molecular approach to the study of distribution and composition of natural bioluminescent dinoflagellate populations. The analysis presented here has resulted in improved insight into the distribution of these organisms in relation to their environment and has highlighted the limitations of optical bioluminescence measurements in studies of bioluminescent dinoflagellates. The greater spatial resolution provided by the molecular approach revealed that hydrographic controls are important in structuring dinoflagellate populations in Patagonian Shelf waters. The application of PCR primers for dinoflagellate *lcf* to map and identify natural populations of bioluminescent dinoflagellates represents a powerful new tool for ecological studies of these organisms.

## Supporting Information

Figure S1 **Circulation at the Patagonian Shelf.** Map of the Patagonian Shelf with general bathymetry; gradient from darkest brown to darkest blue signifying the increasing depth from approximately 100 m to more than 2000 m. The routes of major currents and the areas where their interactions cause well known features such as the shelf break front (SBF) and the Brazil Falklands Currents confluence zone. The SBF becomes sharper moving northward, coinciding with steepening of the shelf break (sharp transition from brown to blue in the bathymetry), and so south of approximately 47°S it covers a less well defined and wider area than depicted by the black line.
(TIF)

Figure S2 **Example of a bioluminescence measurement with the Glowtracka photometer.** The voltage was logged at 1 KHz resolution. This sample was taken at Station 1 at a depth of 4 m and the corresponding blank measurement is shown. The sample was released after approximately 5 seconds (i.e. 5000 milliseconds). When the sample flowed through the detection chamber, high voltage corresponding to the bioluminescence was recorded relative to the blank. After approximately 11 seconds most of the sample had completed its passage through the detection chamber while small amounts were still draining. The measurement was complete after 15 seconds. The raw voltage was converted to photons cm^−2^ s^−1^ by applying the following equation supplied by the manufacturer (Chelsea Technologies, U.K.): Intensity at 560 nm (Megaphotons cm^−2^ s^−1^)  =  (11.570×10^4^× Volts).
(TIF)

Figure S3 **Detection of the dinoflagellate luciferase gene in natural samples.** Gel photograph of the luciferase gene PCR on samples collected during the COPAS cruise, showing the very specific and efficient amplification of the gene from mixed plankton community DNA samples. The first lane in each row is a 50 bp DNA marker and last two lanes are positive and negative control respectively. The 270 bp band marked by an arrow corresponds to the luciferase gene PCR product. Samples are in order of collection i.e. consecutive stations with chlorophyll maximum depth sample first followed by the surface sample.
(TIF)

Table S1 **Data generated in this study.** For each station we show the bioluminescence intensity (BL), detection of the luciferase gene (*lcf*) and cell counts of the various dinoflagellate (dinos) groups and diatoms. As only surface chlorophyll values are shown in the main manuscript, we include the full data set for our stations here.
(DOCX)
