# Supplementary figures and images for: Molecular Detection of Bioluminescent Dinoflagellates in Surface Waters of the Patagonian Shelf during Early Austral Summer 2008
Source: PLoS One. 2014 Jun 11;9(6):e98849. doi: 10.1371/journal.pone.0098849 (PMC4053353; doi:10.1371/journal.pone.0098849)

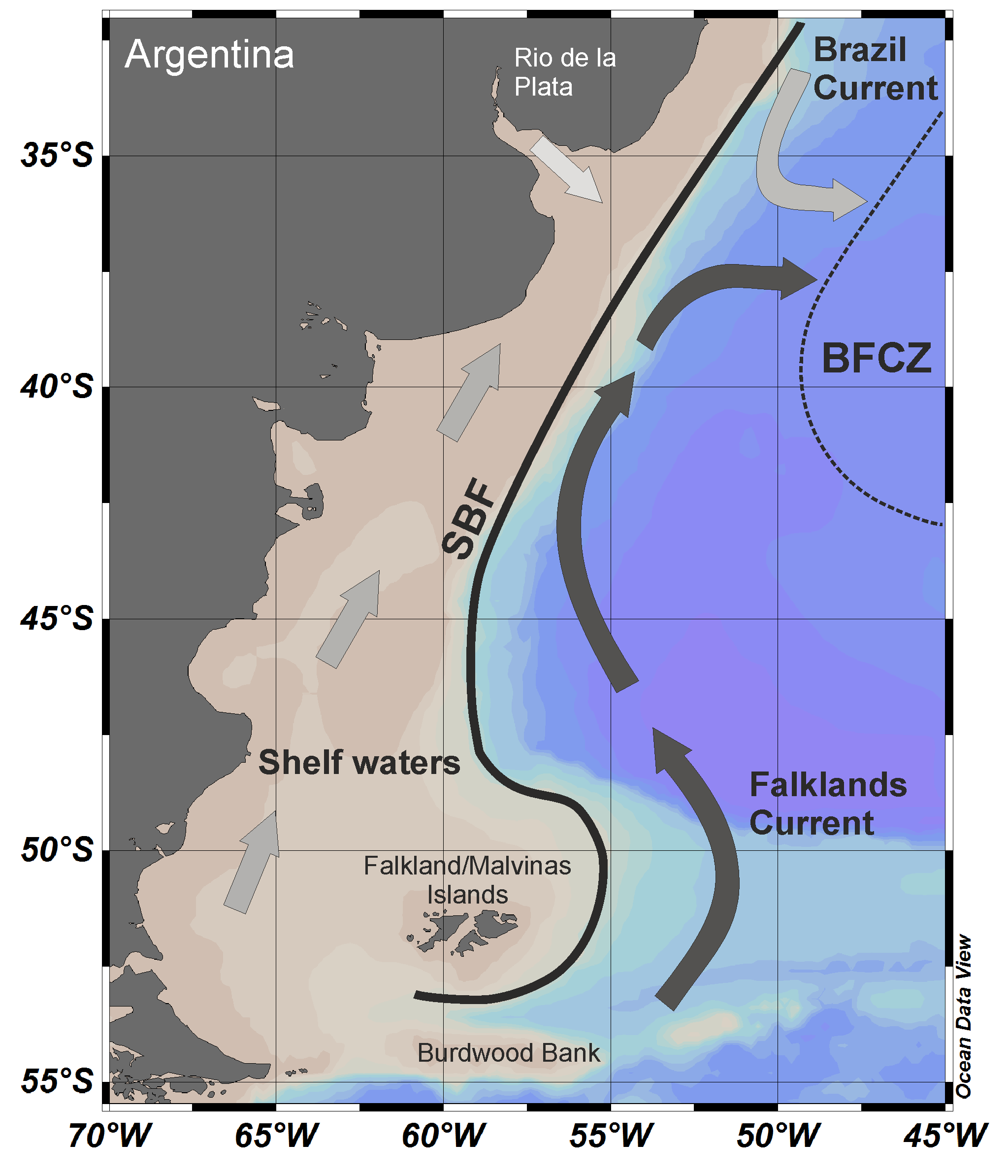

Supplement: Figure S1 — Circulation at the Patagonian Shelf. Map of the Patagonian Shelf with general bathymetry; gradient from darkest brown to darkest blue signifying the increasing depth from approximately 100 m to more than 2000 m. The routes of major currents and the areas where their interactions cause well known features such as the shelf break front (SBF) and the Brazil Falklands Currents confluence zone. The SBF becomes sharper moving northward, coinciding with steepening of the shelf break (sharp transition from brown to blue in the bathymetry), and so south of approximately 47°S it covers a less well defined and wider area than depicted by the black line. (TIF) [file pone.0098849.s001.tif]

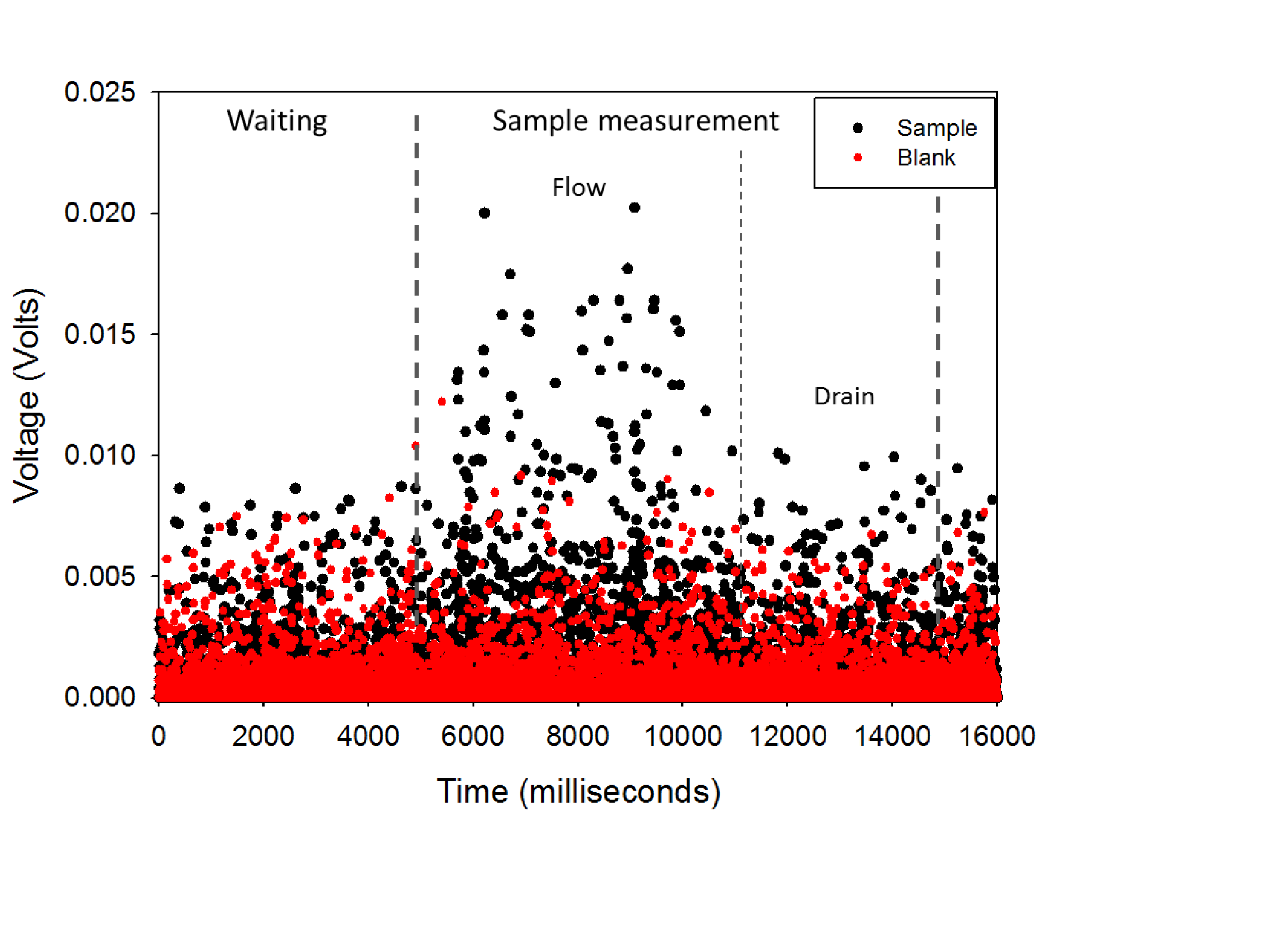

Supplement: Figure S2 — Example of a bioluminescence measurement with the Glowtracka photometer. The voltage was logged at 1 KHz resolution. This sample was taken at Station 1 at a depth of 4 m and the corresponding blank measurement is shown. The sample was released after approximately 5 seconds (i.e. 5000 milliseconds). When the sample flowed through the detection chamber, high voltage corresponding to the bioluminescence was recorded relative to the blank. After approximately 11 seconds most of the sample had completed its passage through the detection chamber while small amounts were still draining. The measurement was complete after 15 seconds. The raw voltage was converted to photons cm−2 s−1 by applying the following equation supplied by the manufacturer (Chelsea Technologies, U.K.): Intensity at 560 nm (Megaphotons cm−2 s−1) = (11.570×104× Volts). (TIF) [file pone.0098849.s002.tif]

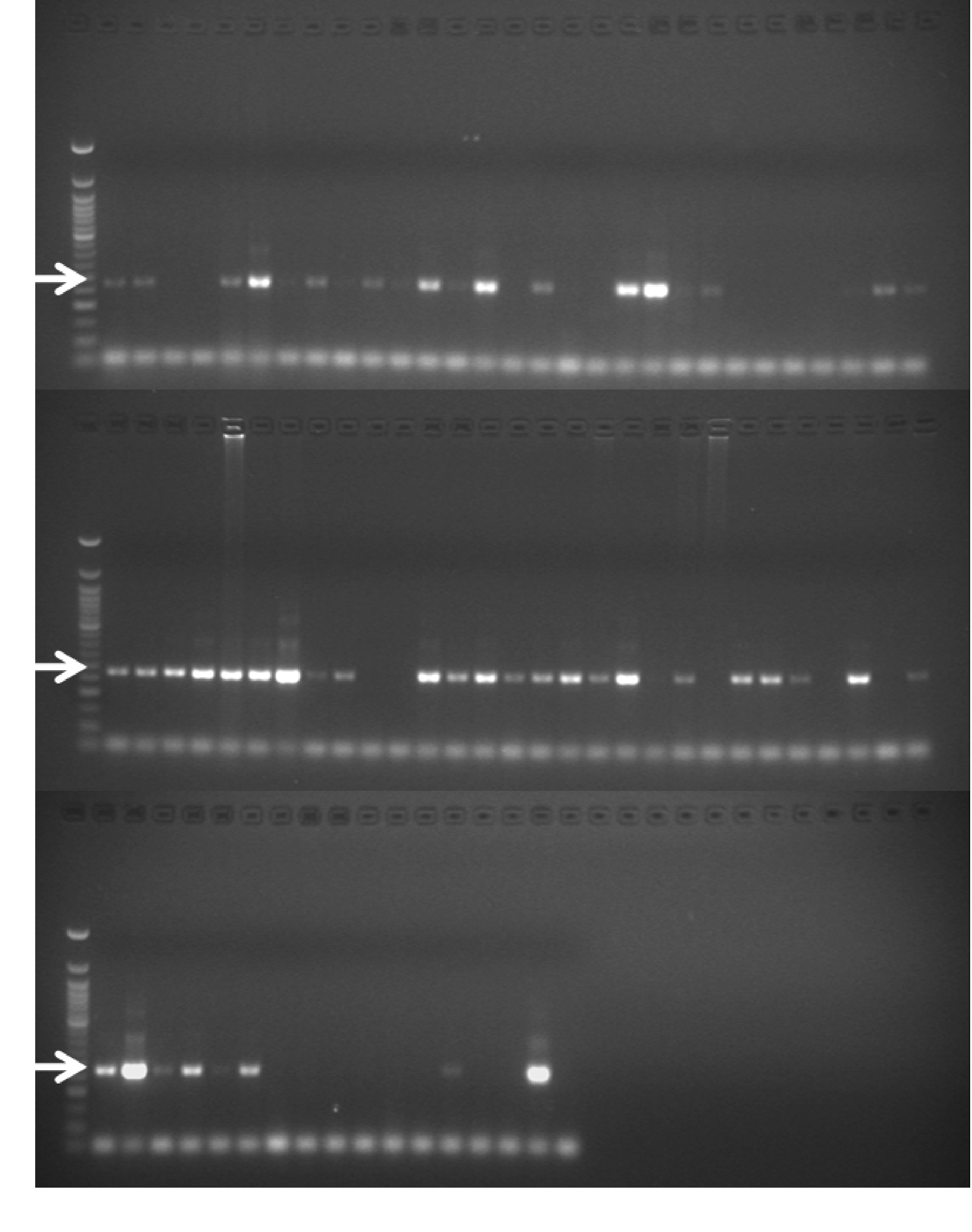

Supplement: Figure S3 — Detection of the dinoflagellate luciferase gene in natural samples. Gel photograph of the luciferase gene PCR on samples collected during the COPAS cruise, showing the very specific and efficient amplification of the gene from mixed plankton community DNA samples. The first lane in each row is a 50 bp DNA marker and last two lanes are positive and negative control respectively. The 270 bp band marked by an arrow corresponds to the luciferase gene PCR product. Samples are in order of collection i.e. consecutive stations with chlorophyll maximum depth sample first followed by the surface sample. (TIF) [file pone.0098849.s003.tif]
