# Supplementary material for: Molecular Detection of Bioluminescent Dinoflagellates in Surface Waters of the Patagonian Shelf during Early Austral Summer 2008
Source: PLoS One. 2014 Jun 11;9(6):e98849. doi: 10.1371/journal.pone.0098849 (PMC4053353; doi:10.1371/journal.pone.0098849)
Supplement: Table S1 — Data generated in this study. For each station we show the bioluminescence intensity (BL), detection of the luciferase gene (lcf) and cell counts of the various dinoflagellate (dinos) groups and diatoms. As only surface chlorophyll values are shown in the main manuscript, we include the full data set for our stations here. (DOCX) [file pone.0098849.s004.docx]

Table S1. Data generated in this study. For each station we show the bioluminescence intensity (BL), detection of the luciferase gene (*lcf*) and cell counts of the various dinoflagellate (dinos) groups and diatoms. As only surface chlorophyll values are shown in the main manuscript, we include the full data set for our stations here.

| **Station** | **Depth (m)** | **Date** | **Time (GMT)** | **Lat (ºS)** | **Long (ºW)** | **Chlorophyll (µg L^-1^)** | **BL (photons cm^-2^ s-1)** | ***lcf*** | **BL dinos (cells mL^-1^)** | **Non BL dinos (cells mL^-1^)** | **Total dinos (cells mL^-1^)** | **Diatoms (cells mL^-1^)** |
| --- | --- | --- | --- | --- | --- | --- | --- | --- | --- | --- | --- | --- |
| 1 | 5.6 | 20081205 | 11:58 | 37.33 | 55.75 |  | 4.40E+11 | Yes |  |  |  |  |
| 1 | 19.5 | 20081205 | 12:26 | 37.33 | 55.75 | 0.65 | 8.05E+11 | Yes |  |  |  |  |
| 3 | 4.4 | 20081205 | 1:04 | 37.92 | 54.50 | 0.67 | 3.07E+11 | No |  |  |  |  |
| 3 | 18.4 | 20081205 | 1:16 | 37.92 | 54.50 | 0.37 | 1.71E+11 | No |  |  |  |  |
| 5 | 6.7 | 20081206 | 7:22 | 38.13 | 54.07 | 2.29 | 3.31E+11 | Yes | 120 | 13716 | 13836 | 121480 |
| 5 | 16.5 | 20081206 | 7:33 | 38.13 | 54.07 | 2.71 | 4.40E+11 | Yes | 0 | 1384 | 1384 | 34364 |
| 7 | 4.5 | 20081206 | 20:43 | 38.49 | 53.28 | 0.34 | 1.99E+11 | Yes |  |  |  |  |
| 7 | 22.4 | 20081206 | 21:17 | 38.49 | 53.28 |  | 1.66E+11 | Yes |  |  |  |  |
| 7 | 53.5 | 20081206 | 21:32 | 38.49 | 53.28 | 0.58 | 5.89E+10 | No |  |  |  |  |
| 8 | 3 | 20081207 | 6:12 | 38.77 | 52.63 | 0.14 | 2.20E+11 | Yes |  |  |  |  |
| 8 | 64.8 | 20081207 | 6:36 | 38.77 | 52.63 | 0.43 | 1.75E+11 | No |  |  |  |  |
| 10 | 6.9 | 20081207 | 5:17 | 39.40 | 51.42 |  | 3.06E+11 | Yes | 340 | 5220 | 5560 | 992 |
| 10 | 36.7 | 20081207 | 5:28 | 39.40 | 51.42 |  | 2.02E+11 | Yes | 480 | 2320 | 2800 | 120 |
| 10.2 | 6.9 | 20081208 | 20:37 | 39.40 | 51.42 | 0.39 | 1.83E+11 | Yes |  |  |  |  |
| 10.2 | 42.4 | 20081208 | 21:08 | 39.40 | 51.42 | 0.54 | 1.68E+11 |  |  |  |  |  |
| 13 | 3.7 | 20081209 | 2:02 | 41.13 | 54.45 | 0.54 | 1.26E+11 |  |  |  |  |  |
| 13 | 26.4 | 20081209 | 2:55 | 41.13 | 54.45 | 0.77 | 1.67E+11 |  |  |  |  |  |
| 15 | 6.9 | 20081209 | 16:01 | 42.27 | 56.45 | 0.76 | 1.64E+11 | No |  |  |  |  |
| 15 | 21.9 | 20081209 | 16:18 | 42.27 | 56.45 | 0.70 | 2.10E+11 | Yes |  |  |  |  |
| 16 | 2.6 | 20081210 | 1:37 | 42.81 | 57.47 | 1.10 | 1.88E+11 |  | 0 | 7540 | 7540 | 7956 |
| 16 | 19.1 | 20081209 | 1:10 | 42.81 | 57.47 | 1.07 | 1.53E+11 | Yes | 0 | 1524 | 1524 | 10628 |
| 17 | 3 | 20081210 | 5:24 | 43.37 | 58.46 | 1.42 | 1.31E+11 | Yes | 120 | 12200 | 12320 | 17900 |
| 17 | 18.7 | 20081210 | 5:24 | 43.37 | 58.46 | 1.45 | 1.64E+11 | Yes | 280 | 5000 | 5280 | 18820 |
| 18 | 2.9 | 20081210 | 16:42 | 43.92 | 59.49 | 0.81 | 1.83E+11 | Yes | 40 | 326870 | 326910 | 720 |
| 18 | 52.3 | 20081210 | 17:07 | 43.92 | 59.49 | 1.66 | 1.29E+11 | No | 120 | 370540 | 370660 | 280 |
| 19 | 2.6 | 20081210 | 21:00 | 44.47 | 60.51 | 0.52 |  | No |  |  |  |  |
| 19 | 34.8 | 20081210 | 21:00 | 44.47 | 60.51 | 1.17 |  | No |  |  |  |  |
| 20 | 4.3 | 20081211 | 4:30 | 45.00 | 61.50 | 0.75 | 1.92E+11 | No |  |  |  |  |
| 20 | 25.2 | 20081211 | 4:17 | 45.00 | 61.50 | 0.11 | 1.84E+11 | No |  |  |  |  |
| 24 | 4.4 | 20081211 | 23:45 | 45.00 | 59.71 | 0.80 | 1.81E+11 | Yes | 0 | 36600 | 36600 | 840 |
| 24 | 50 | 20081211 | 23:59 | 45.00 | 59.71 | 0.75 | 1.59E+11 | No | 200 | 455592 | 455792 | 960 |
| 32 | 4.6 | 20081213 | 7:17 | 46.25 | 57.00 | 0.91 | 1.44E+11 | Yes | 80 | 8200 | 8280 | 3160 |
| 32 | 24.8 | 20081213 | 7:33 | 46.25 | 57.00 | 0.84 | 1.21E+11 | Yes | 40 | 4440 | 4480 | 1080 |
| 37 | 2.4 | 20081213 | 23:43 | 46.25 | 59.81 | 0.57 | 2.27E+11 | Yes |  |  |  |  |
| 38 | 2.1 | 20081214 | 4:15 | 46.25 | 60.38 | 0.50 | 2.34E+11 | Yes | 380 | 41876 | 42256 | 20 |
| 38 | 25.1 | 20081214 | 4:25 | 46.25 | 60.38 | 0.51 | 2.13E+11 | Yes | 180 | 57808 | 57988 | 0 |
| 46 | 5.6 | 20081214 | 22:33 | 47.50 | 60.38 | 3.89 | 2.37E+11 | Yes | 1040 | 2195648 | 2196688 | 0 |
| 47 | 3.4 | 20081215 | 2:47 | 47.49 | 60.22 | 1.10 | 2.91E+11 | Yes | 4400 | 12680 | 17080 | 9000 |
| 47 | 34.6 | 20081215 | 3:55 | 47.49 | 60.22 | 1.45 | 0.00E+00 | Yes | 1000 | 3120 | 4120 | 9840 |
| 52 | 5.6 | 20081216 | 5:40 | 47.50 | 57.00 | 2.14 | 1.02E+11 | Yes | 120 | 9160 | 9280 | 680 |
| 52 | 28.7 | 20081216 | 6:01 | 47.50 | 57.00 | 0.97 | 2.05E+11 | Yes | 400 | 11840 | 12240 | 280 |
| 58 | 4.6 | 20081216 | 22:46 | 48.75 | 58.11 | 0.83 | 9.30E+10 | No |  |  |  |  |
| 58 | 29.5 | 20081216 | 22:55 | 48.75 | 58.11 | 1.11 | 2.49E+11 | No |  |  |  |  |
| 59.5 | 13 | 20081217 | 4:15 | 48.75 | 58.66 |  | 1.76E+11 | Yes |  |  |  |  |
| 60 | 6.9 | 20081217 | 6:21 | 48.75 | 59.16 | 2.54 | 2.05E+11 | Yes | 780 | 106360 | 107140 | 4520 |
| 60 | 12.9 | 20081217 | 6:30 | 48.75 | 59.16 | 1.41 | 1.27E+11 | Yes | 200 | 37280 | 37480 | 3600 |
| 66 | 3.7 | 20081218 | 4:23 | 49.30 | 62.16 | 0.97 | 2.50E+11 | Yes |  |  |  |  |
| 66 | 18.6 | 20081218 | 5:31 | 49.30 | 62.16 | 0.86 | 1.63E+11 |  |  |  |  |  |
| 68 | 5.2 | 20081218 | 10:36 | 49.63 | 62.79 | 0.26 | 2.24E+11 | Yes | 340 | 15800 | 16140 | 20 |
| 68 | 35.7 | 20081218 | 10:46 | 49.63 | 62.79 | 0.82 | 1.35E+11 | Yes | 40 | 1820 | 1860 | 0 |
| 70 | 6.5 | 20081218 | 22:22 | 49.75 | 61.59 | 0.58 | 1.20E+11 | Yes |  |  |  |  |
| 70 | 43.8 | 20081218 | 22:36 | 49.75 | 61.59 | 2.21 | 1.42E+11 | Yes |  |  |  |  |
| 72 | 3.9 | 20081219 | 4:08 | 49.75 | 60.47 | 0.48 | 1.34E+11 | Yes | 40 | 1694538 | 1694578 | 300 |
| 72 | 27 | 20081219 | 4:24 | 49.75 | 60.47 | 1.46 | 1.36E+11 |  | 80 | 132676 | 132756 | 120 |
| 73 | 5.9 | 20081219 | 6:16 | 49.75 | 59.90 |  | 1.16E+11 | No | 60 | 1928372 | 1928432 | 0 |
| 74 | 4.9 | 20081219 | 8:58 | 49.75 | 59.33 | 1.72 | 1.85E+11 | Yes | 180 | 81340 | 81520 | 20 |
| 74 | 14.1 | 20081219 | 9:08 | 49.75 | 59.33 | 1.66 | 4.23E+11 | Yes | 180 | 47824 | 48004 | 480 |
| 78 | 26.7 | 20081220 | 5:36 | 49.75 | 57.20 | 1.03 | 2.43E+11 | Yes | 160 | 7976 | 8136 | 43112 |
| 80 | 4.3 | 20081220 | 10:35 | 49.75 | 55.92 | 0.55 | 1.30E+11 | Yes | 200 | 18660 | 18860 | 5240 |
| 80 | 27.1 | 20081220 | 10:45 | 49.75 | 55.92 | 0.88 | 2.60E+11 | No | 0 | 15520 | 15520 | 1280 |
| 86 | 8.1 | 20081221 | 4:43 | 50.97 | 54.77 | 1.57 | 2.15E+11 | No | 2380 | 8720 | 11100 | 531400 |
| 86 | 48.1 | 20081221 | 5:07 | 50.97 | 54.77 | 1.51 | 1.17E+11 | Yes | 100 | 5940 | 6040 | 634040 |
| 100 | 5.3 | 20081223 | 22:20 | 48.13 | 59.72 | 0.53 | 1.76E+11 | Yes |  |  |  |  |
| 100 | 36.3 | 20081223 | 22:27 | 48.13 | 59.72 | 0.69 | 9.37E+09 | Yes | 20 | 680 | 700 | 28440 |
| 102 | 5.2 | 20081224 | 4:44 | 48.75 | 59.23 | 0.34 | 1.89E+11 | Yes |  |  |  |  |
| 102 | 29 | 20081224 | 4:15 | 48.75 | 59.23 | 0.56 | 1.62E+11 | Yes |  |  |  |  |
| 108 | 6 | 20081225 | 4:20 | 50.36 | 56.22 | 0.65 | 1.05E+11 | Yes |  |  |  |  |
| 108 | 41.2 | 20081225 | 4:31 | 50.36 | 56.22 | 0.90 | 1.98E+11 | Yes |  |  |  |  |
| 116 | 4 | 20081226 | 7:54 | 51.97 | 57.62 |  | 1.76E+11 | No |  |  |  |  |
| 116 | 27.8 | 20081226 | 8:21 | 51.97 | 57.62 | 1.91 | 2.70E+11 | No |  |  |  |  |
| 122 | 7.5 | 20081227 | 4:59 | 52.90 | 55.34 | 1.16 | 2.91E+11 | No | 320 | 4000 | 4320 | 13560 |
| 122 | 47.1 | 20081227 | 5:13 | 52.90 | 55.34 | 1.41 | 2.37E+11 | No | 0 | 1320 | 1320 | 16400 |
| 128 | 6.1 | 20081228 | 4:32 | 52.38 | 58.32 | 1.06 | 1.55E+11 | No | 20 | 320 | 340 | 0 |
| 128 | 40.2 | 20081228 | 4:40 | 52.38 | 58.32 | 0.80 | 3.22E+11 | No | 0 | 180 | 180 | 0 |
| 134 | 6.1 | 20081229 | 3:12 | 54.11 | 58.30 | 0.60 | 3.52E+11 | Yes | 100 | 18940 | 19040 | 12840 |
| 142 | 5.4 | 20081230 | 4:52 | 52.61 | 60.28 | 2.02 | 2.54E+11 | No | 0 | 1000 | 1000 | 206500 |
| 142 | 34.5 | 20081230 | 5:00 | 52.61 | 60.28 | 1.85 | 1.93E+11 | No | 20 | 520 | 540 | 172880 |
